# Major challenges in clinical management of TB/HIV co-infected patients in Eastern Europe compared with Western Europe and Latin America

**DOI:** 10.7448/IAS.17.4.19505

**Published:** 2014-11-02

**Authors:** Anne Marie Efsen, Anna Schultze, Frank Post, Alexander Panteleev, Hansjakob Furrer, Robert Miller, Aliaksandr Skrahin, Marcelo H Losso, Javier Toibaro, Enrico Girardi, José Miro, Mathias Bruyand, Niels Obel, Joan Caylá, Daria Podlekareva, Jens Lundgren, Amanda Mocroft, Ole Kirk, EuroCoord TB/HIV Study Group

**Affiliations:** 1 Department of Infectious Diseases and Rheumatology, CHIP, Rigshospitalet, University of Copenhagen, Copenhagen, Denmark; 2 Department of Infection and Population Health, University College London, London, UK; 3 Department of Sexual Health, King's College Hospital, London, UK; 4 City TB Hospital 2, HIV/TB, St. Petersburg, Russian Federation; 5 Department of Infectious Diseases, Bern University Hospital and University of Bern, Bern, Switzerland; 6 Centre for Sexual Health and HIV Research, University College London, London, UK; 7 Republican Research and Practical Centre for Pulmonary and TB, Clinical Department, Minsk, Belarus; 8 Hospital Gral. de Agudos JM Ramos Mejía, Servicio Inmunocomprometidos, Buenos Aires, Argentina; 9 Istituto Nazionale Malattie Infettive L. Spallanzani – IRCCS, UOC Epidemiologia Clinica, Rome, Italy; 10 Hospital Clinic Universitari de Barcelona, Hospital Clinic Universitari, Barcelona, Spain; 11 Université Bordeaux Segalen, INSERM U897, Bordeaux, France

## Abstract

**Introduction:**

Rates of both TB/HIV co-infection and multi-drug-resistant (MDR) TB are increasing in Eastern Europe (EE). Data on the clinical management of TB/HIV co-infected patients are scarce. Our aim was to study the clinical characteristics of TB/HIV patients in Europe and Latin America (LA) at TB diagnosis, identify factors associated with MDR-TB and assess the activity of initial TB treatment regimens given the results of drug-susceptibility tests (DST).

**Material and Methods:**

We enrolled 1413 TB/HIV patients from 62 clinics in 19 countries in EE, Western Europe (WE), Southern Europe (SE) and LA from January 2011 to December 2013. Among patients who completed DST within the first month of TB therapy, we linked initial TB treatment regimens to the DST results and calculated the distribution of patients receiving 0, 1, 2, 3 and ≥4 active drugs in each region. Risk factors for MDR-TB were identified in logistic regression models.

**Results:**

Significant differences were observed between EE (*n*=844), WE (*n*=152), SE (*n*=164) and LA (*n*=253) for use of combination antiretroviral therapy (cART) at TB diagnosis (17%, 40%, 44% and 35%, *p*<0.0001), a definite TB diagnosis (culture and/or PCR positive for *Mycobacterium tuberculosis*; 47%, 71%, 72% and 40%, *p*<0.0001) and MDR-TB prevalence (34%, 3%, 3% and 11%, *p* <0.0001 among those with DST results). The history of injecting drug use [adjusted OR (aOR) = 2.03, (95% CI 1.00–4.09)], prior TB treatment (aOR = 3.42, 95% CI 1.88–6.22) and living in EE (aOR = 7.19, 95% CI 3.28–15.78) were associated with MDR-TB. For 569 patients with available DST, the initial TB treatment contained ≥3 active drugs in 64% of patients in EE compared with 90–94% of patients in other regions (Figure 1a). Had the patients received initial therapy with standard therapy [Rifampicin, Isoniazid, Pyrazinamide, Ethambutol (RHZE)], the corresponding proportions would have been 64% vs. 86–97%, respectively (Figure 1b).

**Conclusions:**

In EE, TB/HIV patients had poorer exposure to cART, less often a definitive TB diagnosis and more often MDR-TB compared to other parts of Europe and LA. Initial TB therapy in EE was sub-optimal, with less than two-thirds of patients receiving at least three active drugs, and improved compliance with standard RHZE treatment does not seem to be the solution. Improved management of TB/HIV patients requires routine use of DST, initial TB therapy according to prevailing resistance patterns and more widespread use of cART.

**Figure 1:**
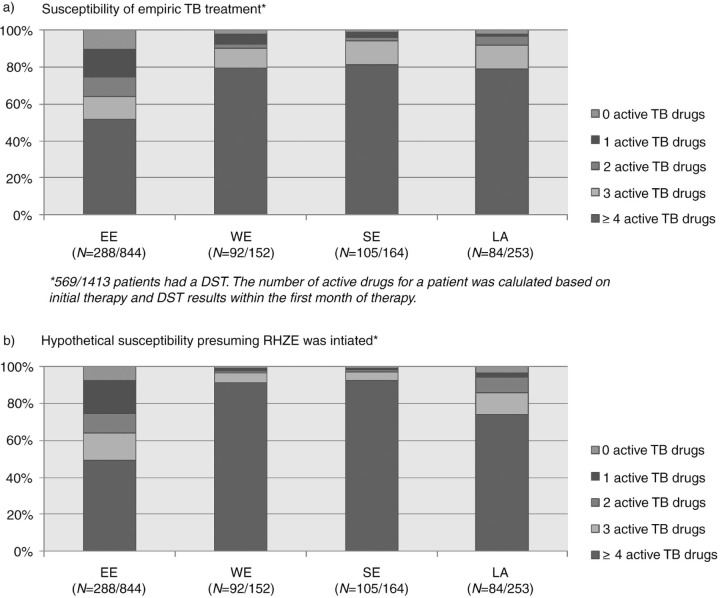
Susceptibility of initial TB treatment.

